# SARS-CoV-2 Infection in One Cat and Three Dogs Living in COVID-19-Positive Households in Madrid, Spain

**DOI:** 10.3389/fvets.2021.779341

**Published:** 2021-11-10

**Authors:** Guadalupe Miró, Javier Regidor-Cerrillo, Rocio Checa, Carlos Diezma-Díaz, Ana Montoya, Jesús García-Cantalejo, Pedro Botías, Javier Arroyo, Luis-Miguel Ortega-Mora

**Affiliations:** ^1^Pet Parasite Lab, Department of Animal Health, Faculty of Veterinary Sciences, Complutense University of Madrid, Madrid, Spain; ^2^Saluvet-Innova S.L., Faculty of Veterinary Sciences, Complutense University of Madrid, Madrid, Spain; ^3^Unidad de Genómica, Centro de Asistencia a la Investigación-Técnicas Biológicas, Complutense University of Madrid, Madrid, Spain; ^4^Saluvet, Department of Animal Health, Faculty of Veterinary Sciences, Complutense University of Madrid, Madrid, Spain

**Keywords:** cat, dog, SARS-CoV-2 infection, COVID-19-positive households, Spain, alpha variant

## Abstract

In this study, we describe SARS-CoV-2 infection dynamics in one cat and three dogs from households with confirmed human cases of COVID-19 living in the Madrid Community (Spain) at the time of expansion (December 2020 through June 2021) of the alpha variant (lineage B.1.1.7). A thorough physical exam and nasopharyngeal, oropharyngeal, and rectal swabs were collected for real-time reverse-transcription PCR (RT-qPCR) SARS-CoV-2 testing on day 0 and in successive samplings on days 7, 14, 21, and 47 during monitoring. Blood was also drawn to determine complete blood counts, biochemical profiles, and serology of the IgG response against SARS-CoV-2. On day 0, the cat case 1 presented with dyspnea and fever associated with a mild bronchoalveolar pattern. The dog cases 2, 3, and 4 were healthy, but case 2 presented with coughing, dyspnea, and weakness, and case 4 exhibited coughing and bilateral nasal discharge 3 and 6 days before the clinical exam. Case 3 (from the same household as case 2) remained asymptomatic. SARS-CoV-2 detection by RT-qPCR showed that the cat case 1 and the dog case 2 exhibited the lowest cycle threshold (Ct) (Ct < 30) when they presented clinical signs. Viral detection failed in successive samplings. Serological analyses revealed a positive IgG response in cat case 1 and dog cases 3 and 4 shortly after or simultaneously to virus shedding. Dog case 2 was seronegative, but seroconverted 21 days after SARS-CoV-2 detection. SARS-CoV-2 genome sequencing was attempted, and genomes were classified as belonging to the B.1.1.7 lineage.

## Introduction

Severe acute respiratory syndrome coronavirus 2 (SARS-CoV-2) was first detected in Wuhan in December 2019. On January 31, 2020, the coronavirus outbreak was declared a public health emergency of international concern by the World Health Organization (1). To date, ~200 million humans have determined to be infected worldwide, and more than 4 million deaths have occurred (2, 3). Between December 2020 and June 2021, the third and fourth epidemic periods of COVID-19 occurred in Spain, coinciding with the expansion of the alpha variant of SARS-CoV-2 (corresponding to lineage B.1.1.7) that spread throughout Europe and North America during the first months of 2021 until it became the dominant variant. By the end of June 2021, this variant had reached levels between 80 and 100% in all regions of Spain (4).

Despite the zoonotic origin of SARS-CoV-2, the role of animals in the COVID-19 pandemic is still being investigated. As the pandemic progresses, the discovery of additional animal species infected with SARS-CoV-2 has increased, including companion animals (such as dogs, cats, and pet ferrets), captive animals (tigers, lions, gorillas, otters, pumas, and snow leopards) and minks from mink farms in America, Asia, and Europe (OIE, 2021). To date, all cases described in animals have been associated with inverse zoonosis (human-to-animal transmission) from zookeepers, mink farmworkers, or owners living with dogs and cats (5, 6).

To date, the total number of PCR-positive domestic cats and dogs worldwide is 102 and 86, respectively (7). However, only a low percentage of viral detection (<10%) has been confirmed by sequencing (8–13). In this study, we describe the SARS-CoV-2 infection dynamics in three dogs (two with mild respiratory signs) and one cat (with severe secondary pneumonia) from households with confirmed human cases of COVID-19 living in the Madrid Region (Spain) at the time of expansion of the alpha variant of SARS-CoV-2 (lineage B.1.1.7).

## Materials and Methods

*Epidemiological information, clinical examination, and sampling*. In the cases included in this study, one cat (case 1, Chester) and three dogs (case 2, Trasto; case 3, Bella; and case 4, Bull) were recruited through a collaborative veterinary clinical network from the Madrid Region, including the Veterinary Clinical Hospital of the University Complutense of Madrid (VCH-UCM), between December 2020 and June 2021. Owner consent was obtained in all cases, and this study was performed in accordance with the Spanish Animal Protection laws and International Guiding Principles for Biomedical Research Involving Animals issued by the Council for International Organizations of Medical Science. A thorough physical exam and nasopharyngeal, oropharyngeal, and rectal/conjunctival swabs were collected for SARS-CoV-2 testing in inactivating virus transport and preservation medium (Biocomma, Durviz, Valencia, Spain). In addition, blood samples were drawn by cephalic venepuncture (1.5 ml) for serological testing, complete blood counts (CBCs), and biochemical profiles. Cat case 1 was sedated before sampling. Swab samples were maintained at −20°C, and blood samples were maintained at 4°C until submission to the laboratory for analyses. Once a positive result was detected, further clinical examinations were performed, and samples (oropharyngeal/nasopharyngeal and rectal swabs plus blood samples) were collected 7, 14, 21, or 47 days after the first sampling to perform diagnostic procedures: real-time reverse-transcription PCR (RT-qPCR), serology, CBC, and biochemical analyses.

*CBC and biochemical profiles*. All hematological and biochemical tests were performed at IDEXX laboratories (Madrid, Spain). CBC consisted of red blood cell count, hematocrit, hemoglobin concentration, red cell distribution width, mean corpuscular volume, mean corpuscular hemoglobin, mean corpuscular hemoglobin concentration, leukocyte count, and platelet count. Biochemical profiles included serum concentrations of glucose, total protein, albumin, globulin, urea, creatinine, SDMA, triglycerides, cholesterol, ions (Ca, Na, K, and Cl), and acute phase proteins (APP) such as CRP and haptoglobin, aspartate aminotransferase activity, alanine aminotransferase (ALT) activity, creatine kinase, and alkaline phosphatase.

*SARS-CoV-2 detection*. Collected swabs were immediately processed or stored at −80°C until SARS-CoV-2 detection by RT-qPCR. Viral RNA was obtained from 200 μl of swab transport medium using the Maxwell® RSC Viral Total Nucleic Acid Purification Kit for automated extraction in a Maxwell® RSC 48 Instrument (Promega, Madrid, Spain). Viral RNA detection was performed using the multiplex TaqPath™ COVID-19 CE-IVD RT-PCR Kit targeting SARS-CoV-2 *orf1-ab, S* and *N* genes (Applied Biosystems, Spain) using a QuantStudio 5 Real-Time PCR System. Samples were considered positive when at least two of the three targets were amplified with cycle threshold (Ct) values <37 according to the manufacturer's instructions. Results generated using interpretative software were also confirmed by PCR curve visualization in QuantStudio^TM^ Design and Analysis Software.

*SARS-CoV-2 sequencing*. SARS-CoV-2 genome sequencing was attempted by RT-qPCR using RNA extracted for viral detection. DNA libraries were prepared from 30 to 80 ng of total RNA, following the Illumina RNA Prep with Enrichment (L) Tagmentation protocol (https://emea.support.illumina.com/content/dam/illumina-support/documents/documentation/chemistry_documentation/illumina_prep/RNA/illumina-rna-prep-reference-guide-1000000124435-03.pdf) using the Respiratory Virus Oligo Panel v2 (Illumina, San Diego, CA, USA). This panel targets and characterizes ~40 common respiratory viruses, including SARS-CoV-2. The prepared libraries were equally pooled based on evaluation by Qubit 2.0 and Bioanalyzer 2100 and sequenced on the MiSeq platform (Illumina, San Diego, CA, USA) with 2 × 150 bp paired-end reads. Sequence processing, assembly, and analysis for variant detection were performed using Genome Detective Virus Tool online software (https://www.genomedetective.com/). Clade assignment and phylogenetic visualization were determined using Nextstrain online software (https://clades.nextstrain.org/) through the UShER web interface from the University of California, Santa Cruz (https://genome.ucsc.edu/cgi-bin/hgPhyloPlace) (14, 15). Sequence data have been registered in NCBI's Bioproject database (BioProject ID PRJNA766526).

*Serology*. The IgG antibody against the spike-receptor binding domain (S-RBD)-SARS-CoV-2 in serum samples was tested using enzyme-linked immunosorbent assay (ELISA) that was performed as previously described by Zhao et al. (16) with modifications. Fifty microliters of coating buffer containing S-RBD grown in HEK293 mammalian cells (transients) from the Institute for Protein Design, Seattle, WA (2 μg/ml) was used to coat the microtiter plates (ImmunoPlate Maxisorp, Nunc, Roskilde, Denmark) overnight at 4°C. After three washes with phosphate-buffered saline containing 0.05% Tween 20 (PBST), blocking was performed with PBST containing BSA 2% for 2 h at room temperature (RT), followed by three washes. Next, blocking solution was incubated with 50 μl of dog or cat sera diluted 1:80 for 1 h at 37°C. The specific IgG response was revealed using conjugated anti-recombinant Protein A/G-HRP (Thermo Fisher Scientific, Waltham, Massachusetts, EEUU) diluted 1:20,000 in PBST for dog sera and Peroxidase AffiniPure Goat Anti-Cat IgG (H+L) conjugate (Jackson ImmunoResearch; Cambridge, UK) diluted 1:80,000 in PBST for cat sera. The bound antibodies were detected using 2,2′-zino-bis (3-ethylbenzothiazoline-6-sulfonic) acid substrate (Merck, Darmstadt, Germany). Absorbance was measured at 405 nm using a microplate reader (Multiscan RC 6.0, Labsystems). Positive samples were identified as follows: OD-positive > Average OD-negative animals + (3 × Standard deviation). The average number of OD-negative animals was calculated from a panel of 50 sera samples from dogs and 25 sera samples from cats obtained prior to the pandemic.

## Results

The clinicopathological findings are shown in Table 1, and the RT-qPCR and serological results are shown in Table 2. SARS-CoV-2 infection was detected by RT-qPCR in cat and dog cases by amplification of at least two targets with Cts < 37 on day 0 (Table 2). Amplification of the three targets was achieved from nasopharyngeal swabs from cat case 1 and dog case 2 and from the oropharyngeal swab of dog case 3. S gene detection failed on the remaining dog case 4. The lowest Cts were observed in samples from cat case 1 and dog case 2 (Cts < 30) when they presented, or recently presented clues of clinical signs associated with viral infection. Another healthy male cat (same breed and age) living in the same household as cat case 1 was tested by RT-qPCR as negative. The shedding of SARS-Cov-2 virus was lower (Cts > 30) in dog case 3, which cohabitated with case 2 and was asymptomatic, and dog case 4 (Table 1).

**Table 1:** Animal demography and clinical findings of one cat and three dogs with SARS-CoV-2 infection.

**Case**	**Animal demography**	**Clinical signs showed**	**Clinicopathological abnormalities**	**Onset of infection (owner-pet)**
**1**	Species: **Cat “Chester”** Age: 4 years old Breed: Norwegian Forest crossbreed, Sex: neutered male Indoor cat Location: NE Madrid	**Symptomatic**: Dyspnea and fever Thoracic x-ray: mild bronchoalveolar pattern	Hyperproteinemia 8.4 g/dl Haptoglobin 41.5 mg/dl Triglycerides increased 109 mg/dl	18 days
**2**	Species: **Dog “Trasto”** Age: 2 years old Breed: crossbreed (German shepherd and Weimaraner) Sex: neutered male Location: SW Madrid	**Symptomatic**: Coughing, dyspnea, and weakness	None	3 days
**3**	Species: **Dog “Bella”** Age: 2 years old Breed: crossbreed (German shepherd and Weimaraner) Sex: sterilized female Location: SW Madrid	**Asymptomatic**	Triglycerides 590 mg/dl	3 days
**4**	Species: **Dog “Bull”** Age: 15 years-old Breed: Yorkshire Terrier Sex: neutered male Location: S Madrid	**Symptomatic**: Coughing and bilateral nasal discharge	ALT 137 UI/L, Hyperglobulinemia 4.8 g/dl Hypoalbuminemia 2.6 g/dl Alb/Globs ratio altered 0.54 CRP increase 68.2 mg/dl	6 days

**Table 2:** SARS-CoV-2 detection by RT-PCR and serology results.

		**Day 0**				**Day 7**				**Day 21**				**Day 47**			
		**RT-qPCR^#^**		**Serol.^&^**		**RT-qPCR**		**Serol**.		**RT-qPCR**		**Serol**.		**RT-qPCR**		**Serol**.	
**Case**	Sample^*^	**N (Ct)**	**S (Ct)**	**Orf1ab (Ct)**	**OD**	**N (Ct)**	**S (Ct)**	**Orf1ab (Ct)**	**OD**	**N (Ct)**	**S (Ct)**	**Orf1ab (Ct)**	**OD**	**N (Ct)**	**S (Ct)**	**Orf1ab (Ct)**	**OD**
1	NP	**28.72**	**34.52**	**28.90**		-	-	-									
	OP					-	-	-	**0.901**								
	O					-	-	-									
2	NP	**29.49**	37.72	**30.25**						35.70	-	-		-	-	-	
	OP	**24.74**	**26.09**	**25.85**	0.079					-	-	-	**1.151**	-	-	-	**1.500**
	O													-	-	-	
3	NP	**31.16**	**33.92**	**33.34**						-	-	-		-	-	-	
	OP	**33.42**	-	-	**1.288**					-	-	-	**1.011**	-	-	-	**1.216**
	O									-	-	-		-	-	-	
4	NP	**33.90**	-	**35.24**		-	-	-									
	OP				**1.234**	-	-	-	**1.471**								
	O					-	-	-									

* *Sample. NP, nasopharyngeal swab; OP, Oropharyngeal swab; O, Other sample swab—Case 1: rectal and conjunctival swabs; Cases 2, 3 and 4: rectal swab*.

# *Real-time reverse-transcription PCR (RT-qPCR) results. The number is the Ct value for gene N, gene S, and gene Orf1ab. (-), Undetermined. Blank cell, not done. Bold characters highlight RT-qPCR positive virus detection*.

& *Serology (Serol.) results: IgG antibody levels against S protein by ELISA “in house”. OD, Absorbance at 405 nm. Bold characters highlight positive results*.

On day 0, cat case 1 presented with dyspnea and fever. Thoracic x-ray revealed a mild bronchoalveolar pattern. The primary clinicopathological abnormalities observed were hyperproteinemia and an increase in APP (haptoglobin) and triglycerides (Table 1). The cat was treated with amoxicillin plus clavulanic acid (22 mg/kg/BID *per os*) and methylprednisolone (2 mg/kg sc one dose and afterwards 1 mg/kg *per os*) for 1 week. A good clinical response was evident in the cat (case 1) 2 weeks after treatment started. The owner of case 1 was confirmed to have COVID-19 by antigen testing 18 days before the cat's clinical exam and sampling, including respiratory symptoms (dry coughing and respiratory distress), ageusia, and anosmia, which did not require hospitalization. Two out of three people living in the household had a confirmed diagnosis of COVID-19 by antigen testing. Dog case 2 presented with coughing, dyspnea, and weakness 3 days before the clinical exam and SARS-CoV-2 detection, but case 3 remained asymptomatic. Cases 2 and 3 were living in a household where the four cohabitants (parents and children) were COVID-19 positive, as confirmed by antigen testing, and simultaneously, the mother and teenaged son exhibited slight respiratory symptoms compatible with COVID-19. Dog case 4 presented with coughing and bilateral nasal discharge 6 days before the clinical exam and was SARS-CoV-2 positive by RT-qPCR. Simultaneously, all family members exhibited respiratory symptoms with a confirmed positive diagnosis of SARS-CoV-2 infection by antigen testing during the same week. Dog cases 2 and 4 were normal under clinical exam 3 and 6 days later, respectively (Table 1). Body temperature was also normal in both animals at that time.

Serological analyses revealed a positive IgG response in cat case 1 and dog cases 3 and 4 shortly after—a week for cat case 1—or simultaneously to virus shedding, whereas dog case 2 was considered seronegative. Dog case 2 finally seroconverted 21 days after SARS-CoV-2 detection (Table 2), suggesting recent infection without seroconversion. The cat living with cat case 1 was also seropositive, although viral shedding was not detected by RT-qPCR (data not shown).

The short-time persistence of SARS-CoV-2 shedding decayed 1 week after the first positive sample in cat case 1, at which time the animal had clinically recovered, and 21 days after the presentation of clinical signs of COVID-19 in her owner (Table 2). Viral detection also failed in dog case 4 7 days after the first analysis and 14 days after the detection of respiratory signs in their owners. Residual viral RNA detection was observed, 21 days from the first positive detection, in dog case 2, and respiratory symptoms were exhibited by the owners, whereas there was an absence of detection in case 3 (Table 2). Dog case 2 seroconverted to positive on day 21, and all animals remained seropositive throughout this study (Table 2). Based on our results, we can assume a period for viral shedding of longer than 18 days for cat case 1 and longer than 7 days for dog cases 2, 3, and 4.

**Figure 1:**
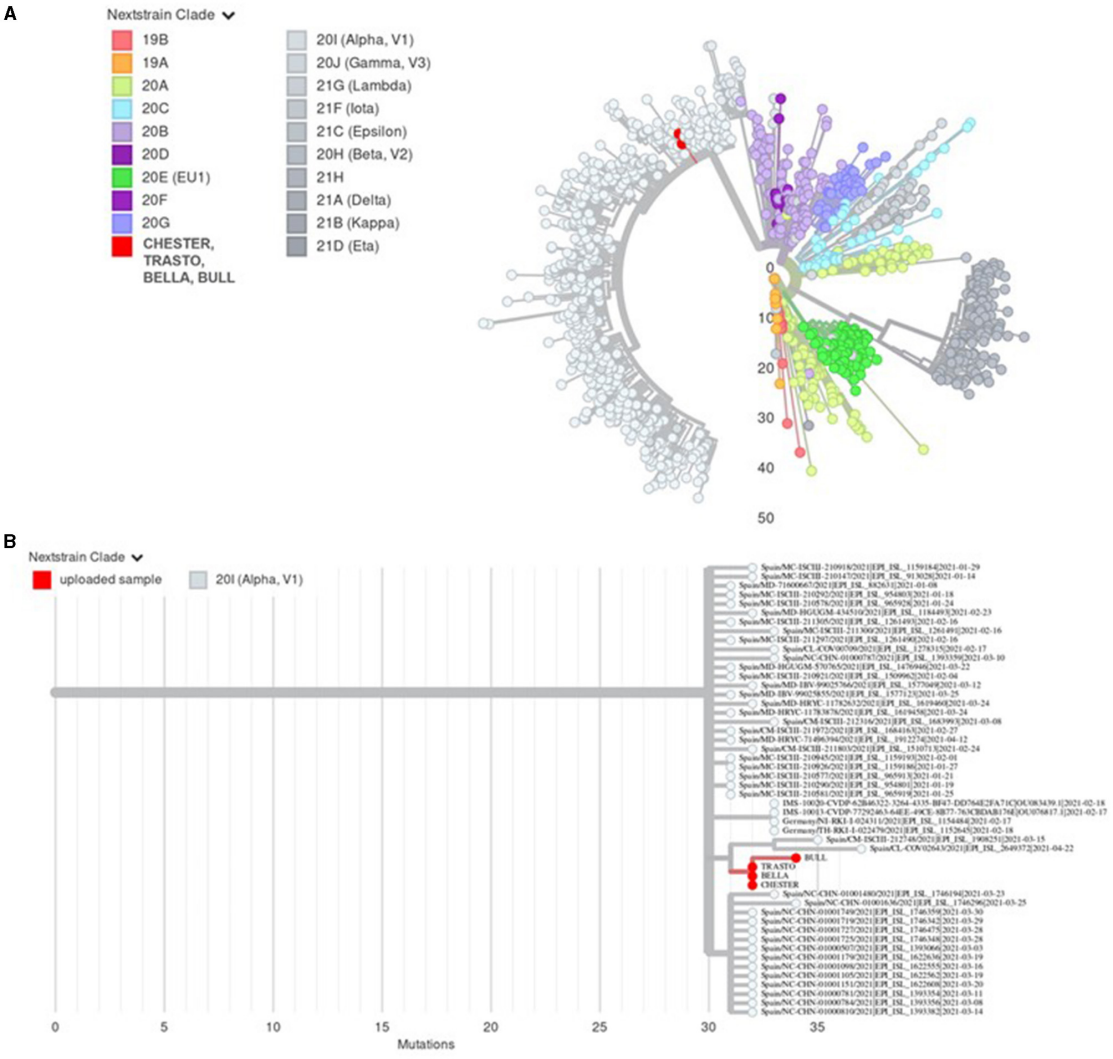
**(A)** Phylogenetic analysis generated by UShER of the four SARS-CoV-2 cases described. The analysis includes 2,664,613 genomes from the GISAID, GenBank, COG-UK, and CNCB databases (updated July 30, 2021). Subtrees including case 1 (Chester), case 2 (Trasto), case 3 (Bella), and case 4 (Bull) sequences plus (a) 1,000 random sequences from the phylogenetic tree for context (radial visualization) or (b) 50 nearest sequences from the phylogenetic tree are shown. **(B)** Sequence comparison showing concordance, nucleic acid, and amino acid changes in the four sequences with respect to the SARS-CoV-2 Reference Sequence as reported in Genome Detective software.

The SARS-CoV-2 genome of all four samples was sequenced. Genomes were compared to the SARS-CoV-2 Genome Reference Sequence isolated from Wuhan (NC_045512.2) (17), demonstrating a sequence identity of 99.7% or higher and a sequence coverage of 79.2, 89.0, 77.1, and 77.1% for cases 1, 2, 3, and 4, respectively. Details of sequence comparisons and changes detected with respect to the SARS-CoV-2 Genome Reference Sequence are shown in Supplementary Table 1. All strains were classified unambiguously as belonging to the B.1.1.7 lineage of Pangolin nomenclature (18, 19), which corresponds to the Next strain Clade 20I (Alpha, VI). The phylogenetic analyses indicated that the four sequences grouped in the same cluster, confirming the lineage and clade classification (Figure 1).

## Discussion

The findings in this study resemble results previously described concerning SARS-CoV-2 detection in pets that were in contact with positive humans. Previous studies demonstrated viral shedding from two cats living with COVID-19 owners in Spain (20, 21) and other countries (5). SARS-CoV-2 detection has been reported in 102 cats around the world at the time of writing this article (OIE, 2021). Experimental infections have demonstrated that SARS-CoV-2 can efficiently replicate in cats and that the virus is transmissible between cats (22). Clinical consequences of COVID-19 in cats include respiratory signs, such as those observed in this study (23), although cats shedding the virus can remain asymptomatic (24–26). The persistence of viral shedding also resembles the time period detected in most naturally infected cats (24, 26) although it can be prolonged as much as 25 days after the first detection (9) and 31 days after estimated SARS-CoV-2 first owner exposition (23).

SARS-CoV-2 infection in domestic dogs from COVID-19 households has also been reported around the world, with 86 declared positive cases (OIE, 2021). To our knowledge, the results in this study describe the first detection of SARS-CoV-2 virus in dogs with clinical signs from Spain. The limited number of reports of natural infection in dogs could be a consequence of their low susceptibility to this infection compared to cats, lower viral titers, and reduced duration of viral shedding, as was determined under experimental infection conditions (22) Naturally infected dogs shedding SARS-CoV-2 virus remain asymptomatic (5). The first reported cases involved a 17-year-old Pomeranian dog from Hong Kong and a 7-year-old male German Shepherd from the US that presented severe clinical signs due to the associated comorbidities (27). Other respiratory pathogens involved in clinical signs were not investigated in this study. Clinical signs were transitory in the dogs included in this study for virus shedding and animals rapidly recovered after virus became undetectable. These clinical outcomes suggest that observed clinical signs were associated with SARS-CoV-2 viral infection. The persistence of viral shedding in naturally infected dogs has been recently studied in eight dogs from Brazil in which viral detection was achieved from 11 to 51 days from the onset of COVID-19 symptoms (28). Viral shedding of the B.1.1.7 variant in one dog from Texas, USA, was detected 1 month after initial detection of infection (29) but viral detection failed in two positive dogs 35 days after owner COVID-19 diagnosis in the same longitudinal study (9).

Serological analyses based on an ELISA using the receptor-binding domain (RBD) of the spike protein as antigen confirmed exposure of pets to SARS-CoV-2 virus. Virus neutralization is accepted as the gold standard of serology detection of antibody response against SARS-CoV-2 virus, but this technique was not performed in this study. In addition, lack of agreement between ELISA and virus neutralization serological techniques could be related with sensibility and specificity characteristics of techniques (9).

Our results support different susceptibilities between dogs and cats naturally exposed to SARS-CoV-2 infection. In contrast to case 1, the cat living in the same household and dog case 3 living with case 2 were clinically healthy during infection. These results are similar to those obtained in Switzerland, where one cat was positive and another was negative living in the same household (10). Another interesting point is the limited time of clinical sign presentation and viral shedding from the estimated exposure to viral infection by the pets in this study. Most cases presented clinical signs simultaneously with their infected owners, and they had seroconverted nearly with their owners' diagnosis, suggesting the same source of infection or a very rapid transmission from the owners to their pets, as previously described (9, 13). Interestingly, all genome sequences identified the B.1.1.7 variant. This alpha variant was predominantly circulating from the first months of 2021 and during the fourth COVID epidemic period (March–June 2021), comprising > 40% of cases in Madrid and reaching 90% of cases identified between July 19 and 25, 2021 (Ministry of Health, Spain). Nevertheless, SARS-CoV-2 virus variant shed by pet owners was not available for sequencing, hampering confirmation of human-to-animal virus transmission. Pet infection by this variant was only reported previously in a dog and a cat from the same household exposed to their infected owner in the US (9).

## Conclusion

Our findings suggest the theory of SARS-CoV-2 infection in cats and dogs from COVID-19-positive households as an inverse zoonosis. The only cat in this study and two out of three RT-qPCR-positive dogs exhibited respiratory clinical signs that were milder in the dog cases. All of them seroconverted shortly after detection of infection in their owners. Viral sequencing in all cases identified the B.1.1.7. variant, which was predominant in human cases in this Spanish region at the time of sampling. Further studies are needed to elucidate how the new variants are changing the epidemiological scenario in household COVID-19 positives with pets.

## Data Availability Statement

The datasets presented in this study can be found in online repositories. The names of the repository/repositories and accession number(s) can be found below: https://www.ncbi.nlm.nih.gov/, BioProject ID PRJNA766526.

## Ethics Statement

The animal study was reviewed and approved by Animal Experimentation Committee-Complutense University of Madrid. Written informed consent was obtained from the owners for the participation of their animals in this study.

## Author Contributions

GM and LMOM proposed and designed the study. JRC and AM collected the samples and participated in the clinical assessment together with GM. CDD and JRC carried out the serological and PCR analysis. JGC, PB, and JA carried out SARS-CoV-2 sequencing. GM, JRC, and LMOM wrote the manuscript, interpreted the results, and were helped by the other co-authors. All authors read and approved the final manuscript.

## Funding

This work was financially supported by the Community of Madrid (Grant Numbers: COV20/01471-CM and COV20/01500-CM). US NIH/NIAID grants and contracts numbers 1 U01 AI151698-01 and HHSN272201700059C.

## Conflict of Interest

The authors declare that the research was conducted in the absence of any commercial or financial relationships that could be construed as a potential conflict of interest.

## Publisher's Note

All claims expressed in this article are solely those of the authors and do not necessarily represent those of their affiliated organizations, or those of the publisher, the editors and the reviewers. Any product that may be evaluated in this article, or claim that may be made by its manufacturer, is not guaranteed or endorsed by the publisher.
