# Supplementary material for: SARS-CoV-2 Infection in One Cat and Three Dogs Living in COVID-19-Positive Households in Madrid, Spain
Source: Front Vet Sci. 2021 Nov 10;8:779341. doi: 10.3389/fvets.2021.779341 (PMC8660077; doi:10.3389/fvets.2021.779341)
Supplement: Supplementary file 1 [file Data_Sheet_1.PDF]

Case 1 - CHESTER

	Begin	End	Coverage	Concordance	Matches	Identities	I/D/M/F	Stop Codons
**NT Sequence**	40	29903	79,20%	99,70%	23688 (99.9%)	23653 (99.8%)	1/10	
Mutations:	241C>T, 841C>T, *913C>T, 3037C>T, *3267C>T, *6954T>C, *11288_11296delTCTGGTTTT, 12400C>T, 14408C>T, 14547G>A, *14676C>T, *15279C>T, *16176T>C, 22103G>C, 23271C>A, *23403A>G, *24506T>G, 24847_24848insT, *24914G>C, *28111A>G, 28273delA, *28111A>C, *28280G>C, 28281A>T, 28282T>A, 28881G>A, 28882G>A, 28883G>C, *28977C>T							
CDS ORF1ab	1	7097	81,80%	99,30%	5804 (99.9%)	5788 (99.7%)	0/3/5/0	1
Protein mutations:	*T1001I (*3267C>T), *I2230T (*6954T>C), *S3675_F3677del (*11288_11296delTCTGGTTTT), P4715L (14408C>T)							
Codon mutations:	GGC192GGT (841C>T), TCC216TCT (913C>T), TTC924TTT (3037C>T), ACT1001ATT (*3267C>T), ATA2230ACA (*6954T>C), TCT3675_TTT3677del (*11288_11296delTCTGGTTTT), CTC4045CTT (12400C>T), CCT4715CTT (14408C>T), AAG4761AAA (14547G>A), CCC4804CCT (*14676C>T), CAC5005CAT (*15279C>T), ACT5304ACC (*16176T>C)							
CDS S	1	1274	76,50%	97,00%	974 (99.9%)	965 (99.0%)	1/0/1/1	1
Protein mutations:	G181R (22103G>C), *A570D (*23271C>A), *D614G (*23403A>G), *S982A (*24506T>G), F1095_V1096insX (24847_24848insT),* D1118H (*24914G>C)							
Codon mutations:	GGA181CGA (22103G>C), GCT570GAT (*23271C>A), GAT614GGT (*23403A>G), TCA982GCA (*24506T>G), TTT1095_GTT1096insT-- (24847_24848insT), GAC1118CAC (*24914G>C)							
CDS ORF3a	1	194	70%	100%	194 (100%)	193 (99.5%)	0/0/0/0	0
CDS E	1	76	100%	100%	76 (100%)	76 (100%)	0/0/0/0	1
CDS M	1	223	73%	99%	163 (100%)	163 (100%)	0/0/0/0	1
CDS ORF6	1	62	100%	100%	62 (100%)	62 (100%)	0/0/0/0	1
CDS ORF7a	1	43	35,20%	97,30%	43 (100%)	42 (97.7%)	0/0/0/0	0
CDS ORF8	66	122	46,70%	96,40%	57 (100%)	56 (98.2%)	0/0/0/0	1
Protein mutations:	*Y73C (*28111A>G)							
Codon mutations:	TAC73TGC (*28111A>G)							
CDS N	1	366	87,10%	98,50%	366 (100%)	361 (98.6%)	0/0/0/0	0
Protein mutations:	*D3L (*28280G>C 28281A>T 28282T>A), R203K (28881G>A 28882G>A), G204R (28883G>C), *S235F (*28977C>T)							
Codon mutations:	GAT3CTA (*28280G>C 28281A>T 28282T>A), AGG203AAA (28881G>A 28882G>A), GGA204CGA							
CDS ORF9b	1	98	100%	100%	98 (100%)	98 (100%)	0/0/0/0	1
CDS ORF14	1	74	100%	98,40%	74 (100%)	73 (98.6%)	0/0/0/0	1
Protein mutations:	G50N (28881G>A 28882G>A 28883G>C)							
Codon mutations:	GGG50AAC (28881G>A 28882G>A 28883G>C)							

Case 2 - TRASTO

	Begin	End	Coverage	Concordance	Matches	Identities	I/D/M/F	Stop Codons
**NT Sequence**	39	29903	77,10%	99,60%	23051 (99.9%)	22994 (99.7%)	1/10	
Mutations:	241C>T, 841C>T, *913C>T, 3037C>T, *3267C>T, *11288_11296delTCTGGTTTT, 12400C>T, 14408C>T, 14547G>A, *14676C>T, *15279C>T, *16176T>C, 22103G>C, *23271C>A, *23403A>G, *24506T>G, 24847_24848insT, *24914G>C, *28111A>G, 28273delA, *28280G>C, 28281A>T, 28282T>A, 28881G>A, 28882G>A, 28883G>C, *28977C>T							
CDS ORF1ab	1	6964	79,30%	98,80%	5623 (99.9%)	5598 (99.5%)	0/3/10/0	0
Protein mutations:	*T1001I (*3267C>T), *S3675_F3677del (*11288_11296delTCTGGTTTT), P4715L (14408C>T)							
Codon mutations:	GGC192GGT (841C>T), TCC216TCT (913C>T), TTC924TTT (3037C>T), ACT1001ATT (*3267C>T), TCT3675_TTT3677del (*11288_11296delTCTGGTTTT), CTC4045CTT (12400C>T), CCT4715CTT (14408C>T), AAG4761AAA (14547G>A), CCC4804CCT (*14676C>T), CAC5005CAT (*15279C>T), ACT5304ACC (*16176T>C)							
CDS S	2	1274	75,30%	94,70%	959 (99.9%)	945 (98.4%)	1/0/6/1	1
Protein mutations:	G181R (22103G>C), *A570D (*23271C>A), *D614G (*23403A>G), *S982A (*24506T>G), F1095_V1096insX (24847_24848insT), *D1118H (*24914G>C)							
Codon mutations:	GGA181CGA (22103G>C), GCT570GAT (*23271C>A), GAT614GGT (*23403A>G), TCA982GCA (*24506T>G), TTT1095_GTT1096insT-- (24847_24848insT), GAC1118CAC (*24914G>C)							
CDS ORF3a	1	188	68%	99%	188 (100%)	187 (99.5%)	0/0/0/0	0
CDS E	1	76	71%	99%	54 (100%)	53 (98.1%)	0/0/0/0	1
CDS M	1	223	73%	99%	163 (100%)	162 (99.4%)	0/0/0/0	1
CDS ORF6	1	62	100%	100%	62 (100%)	62 (100%)	0/0/0/0	1
CDS ORF7a	1	42	34,40%	100,00%	42 (100%)	42 (100%)	0/0/0/0	0
CDS ORF8	66	122	46,70%	96,40%	57 (100%)	56 (98.2%)	0/0/0/0	1
Protein mutations:	*Y73C (*28111A>G)							
Codon mutations:	TAC73TGC (*28111A>G)							
CDS N	1	366	87,10%	98,50%	366 (100%)	361 (98.6%)	0/0/0/0	0
Protein mutations:	*D3L (*28280G>C 28281A>T 28282T>A), R203K (28881G>A 28882G>A), G204R (28883G>C), *S235F (*28977C>T)							
Codon mutations:	GAT3CTA (*28280G>C 28281A>T 28282T>A), AGG203AAA (28881G>A 28882G>A), GGA204CGA							
CDS ORF9b	1	98	100%	100%	98 (100%)	98 (100%)	0/0/0/0	1
CDS ORF14	1	74	100%	98,40%	74 (100%)	73 (98.6%)	0/0/0/0	1
Protein mutations:	G50N (28881G>A 28882G>A 28883G>C)							
Codon mutations:	GGG50AAC (28881G>A 28882G>A 28883G>C)							

Case 3 - BELLA

	Begin	End	Coverage	Concordance	Matches	Identities	I/D/M/F	Stop Codons
**NT Sequence**	39	29903	89,00%	99,80%	26585 (99.9%)	26554 (99.8%)	0/19	
Mutations:	241C>T, 841C>T, *913C>T, 3037C>T, *3267C>T, *5388C>A, *6954T>C, *11288_11296delTCTGGTTTT, 12400C>T, 13571G>T, 14408C>T, 14547G>A, *14676C>T, *15279C>T, *16176T>C, *21766_21771delACATGT, *21992_21994delTAT, 22103G>C, *23063A>T, *23271C>A, *23403A>G, *23709C>T, *24506T>G, *24914G>C, *28111A>G, 28273delA, *28280G>C, 28281A>T, 28282T>A, 28881G>A, 28882G>A, 28883G>C, *28977C>T							
CDS ORF1ab	1	7097	89,60%	99,50%	6356 (99.9%)	6338 (99.7%)	0/3/1/0	1
Protein mutations:	*T1001I (*3267C>T), *A1708D (*5388C>A), *I2230T (*6954T>C), *S3675_F3677del (*11288_11296delTCTGGTTTT), G4436V (13571G>T), P4715L (14408C>T)							
Codon mutations:	GGC192GGT (841C>T), TCC216TCT (913C>T), TTC924TTT (3037C>T), ACT1001ATT (*3267C>T), GCT1708GAT (*5388C>A), ATA2230ACA (*6954T>C), TCT3675_TTT3677del (*11288_11296delTCTGGTTTT), CTC4045CTT (12400C>T), GGT4436GTT (13571G>T), CCT4715CTT (14408C>T), AAG4761AAA (14547G>A), CCC4804CCT (*14676C>T), CAC5005CAT (*15279C>T), ACT5304ACC (*16176T>C)							
CDS S	1	1274	92,50%	98,90%	1176 (99.7%)	1167 (99.0%)	0/3/0/0	1
Protein mutations:	*H69_V70del (*21766_21771delACATGT), *Y144del (*21992_21994delTAT), G181R (22103G>C), *N501Y (*23063A>T), *A570D (*23271C>A),* D614G (*23403A>G), *T716I (*23709C>T), *S982A (*24506T>G), *D1118H (*24914G>C)							
Codon mutations:	ATA68ATC (*21766_21771delACATGT), CAT69_GTC70del (*21766_21771delACATGT), TAT144del (*21992_21994delTAT), GGA181CGA (22103G>C), AAT501TAT (*23063A>T), GCT570GAT (*23271C>A), GAT614GGT (*23403A>G), ACA716ATA (*23709C>T), TCA982GCA (*24506T>G), GAC1118CAC (*24914G>C)							
CDS ORF3a	1	276	100%	100%	276 (100%)	276 (100%)	0/0/0/0	1
CDS E	1	76	100%	100%	76 (100%)	76 (100%)	0/0/0/0	1
CDS M	1	223	100%	100%	223 (100%)	223 (100%)	0/0/0/0	1
CDS ORF6	1	62	100%	100%	62 (100%)	62 (100%)	0/0/0/0	1
CDS ORF7a	1	44	36,10%	98,70%	44 (100%)	43 (97.7%)	0/0/0/0	0
CDS ORF8	66	122	46,70%	96,40%	57 (100%)	56 (98.2%)	0/0/0/0	1
Protein mutations:	*Y73C (*28111A>G)							
Codon mutations:	TAC73TGC (*28111A>G)							
CDS N	1	420	90,50%	98,40%	380 (100%)	375 (98.7%)	0/0/0/0	1
Protein mutations:	*D3L (*28280G>C 28281A>T 28282T>A), R203K (28881G>A 28882G>A), G204R (28883G>C), *S235F (*28977C>T)							
Codon mutations:	GAT3CTA (*28280G>C 28281A>T 28282T>A), AGG203AAA (28881G>A 28882G>A), GGA204CGA							
CDS ORF9b	1	98	100%	100%	98 (100%)	98 (100%)	0/0/0/0	1
CDS ORF14	1	74	100%	98,40%	74 (100%)	73 (98.6%)	0/0/0/0	1
Protein mutations:	G50N (28881G>A 28882G>A 28883G>C)							
Codon mutations:	GGG50AAC (28881G>A 28882G>A 28883G>C)							

Case 4 - BULL

	Begin	End	Coverage	Concordance	Matches	Identities	I/D/M/F	Stop Codons
**NT Sequence**	50	29903	77,10%	99,70%	23049 (99.9%)	23004 (99.7%)	0/13	
Mutations:	241C>T, 841C>T, *913C>T, 3037C>T, *3267C>T, *6954T>C, *11288_11296delTCTGGTTTT, 12400C>T, 14408C>T, 14547G>A, *14676C>T, *15279C>T, *16176T>C, 18885C>T, *21992_21994delTAT, 22103G>C, *23271C>A, *23403A>G, *24506T>G, *24914G>C, *28111A>G, 28273delA, *28280G>C, 28281A>T, 28282T>A, 28881G>A, 28882G>A, 28883G>C, *28977C>T							
CDS ORF1ab	1	7097	79,10%	99,10%	5614 (99.9%)	5590 (99.5%)	0/3/8/0	1
Protein mutations:	*T1001I (*3267C>T), *I2230T (*6954T>C), *S3675_F3677del (*11288_11296delTCTGGTTTT), P4715L (14408C>T)							
Codon mutations:	GGC192GGT (841C>T), TCC216TCT (913C>T), TTC924TTT (3037C>T), ACT1001ATT (*3267C>T), ATA2230ACA (6954T>C), TCT3675_TTT3677del (*11288_11296delTCTGGTTTT), CTC4045CTT (12400C>T), CCT4715CTT (14408C>T), AAG4761AAA (14547G>A), CCC4804CCT (*14676C>T), CAC5005CAT (*15279C>T), ACT5304ACC (*16176T>C), GTC6207GTT (18885C>T)							
CDS S	1	1274	82,70%	98,40%	1053 (99.9%)	1044 (99.1%)	0/1/0/0	1
Protein mutations:	*Y144del (*21992_21994delTAT), G181R (22103G>C), *A570D (*23271C>A), *D614G (*23403A>G), *S982A (*24506T>G),* D1118H (*24914G>C)							
Codon mutations:	TAT144del (*21992_21994delTAT), GGA181CGA (22103G>C), GCT570GAT (*23271C>A), GAT614GGT (*23403A>G), TCA982GCA (*24506T>G), GAC1118CAC (*24914G>C)							
CDS ORF3a	1	199	72%	99%	199 (100%)	198 (99.5%)	0/0/0/0	0
CDS E	61	76	21%	96%	16 (100%)	16 (100%)	0/0/0/0	1
CDS M	1	223	58%	100%	130 (100%)	130 (100%)	0/0/0/0	1
CDS ORF6	1	62	100%	100%	62 (100%)	62 (100%)	0/0/0/0	1
CDS ORF7a	1	45	36,90%	96,10%	45 (100%)	44 (97.8%)	0/0/0/0	0
CDS ORF8	66	122	46,70%	96,40%	57 (100%)	56 (98.2%)	0/0/0/0	1
Protein mutations:	*Y73C (*28111A>G)							
Codon mutations:	TAC73TGC (*28111A>G)							
CDS N	1	416	94,00%	98,40%	395 (100%)	389 (98.5%)	0/0/0/0	0
Protein mutations:	*D3L (*28280G>C 28281A>T 28282T>A), R203K (28881G>A 28882G>A), G204R (28883G>C), *S235F (*28977C>T)							
Codon mutations:	GAT3CTA (*28280G>C 28281A>T 28282T>A), AGG203AAA (28881G>A 28882G>A), GGA204CGA							
CDS ORF9b	1	98	100%	100%	98 (100%)	98 (100%)	0/0/0/0	1
CDS ORF14	1	74	100%	98,40%	74 (100%)	73 (98.6%)	0/0/0/0	1
Protein mutations:	G50N (28881G>A 28882G>A 28883G>C)							
Codon mutations:	GGG50AAC (28881G>A 28882G>A 28883G>C)							

I/D/M/F: Inserts / Deletes / Misaligned / Frameshifts

***: Present in B.1.1.7 strain**
